# Strategies That Promote Equity in COVID-19 Vaccine Uptake for Black Communities: a Review

**DOI:** 10.1007/s11524-021-00594-3

**Published:** 2022-01-11

**Authors:** Debbie Dada, Joseph Nguemo Djiometio, SarahAnn M. McFadden, Jemal Demeke, David Vlahov, Leo Wilton, Mengzu Wang, LaRon E. Nelson

**Affiliations:** 1 Yale College, New Haven, CT USA; 2 St Michael’s Hospital Centre for Urban Health Solutions, Toronto, ON Canada; 3 Yale University School of Nursing, Orange, Campus Office #11501, P.O. Box 27399, West Haven, CT 06516-0972 USA; 4 School of Public Health, Yale University, New Haven, CT USA; 5 State University of New York at Binghamton, Binghamton, NY USA; 6 Faculty of Humanities, University of Johannesburg, Johannesburg, South Africa; 7 Science Applications International Corporation (“SAIC”), Reston, VA USA

**Keywords:** COVID-19, Vaccine, Black community, Equity, Interventions

## Abstract

Black communities have had a high burden of COVID-19 cases, hospitalizations, and death, yet rates of COVID-19 vaccine uptake among Blacks lag behind other demographic groups. This has been due in part to vaccine hesitancy and multi-level issues around access to COVID-19 vaccines. Effective strategies to promote vaccine uptake among Black communities are needed. To perform a rapid review covering December 2020–August 2021, our search strategy used PubMed, Google, and print media with a prescribed set of definitions and search terms for two reasons: there were limited peer-reviewed studies during the early period of vaccine roll-out and real-time perspectives were crucially needed. Analyses included expert opinion, descriptions of implemented projects, and project outcomes. The strategies described in these reports largely converged into three categories: (a) addressing mistrust, (b) combatting misinformation, and (c) improving access to COVID-19 vaccines. When working to reduce hesitancy, it is important to consider messaging content, messengers, and location. To address mistrust, reports detailed the importance of communicating through trusted channels, validating the real, history- and experience-based reasons why people may be hesitant to establish common ground, and addressing racism embedded within the healthcare system. To combat misinformation, strategies included dispelling myths and answering questions through town halls and culturally intelligent outreach. Black physicians and clinicians are considered trusted messengers and partnering with community leaders such as pastors can help to reach more people. The settings of vaccination sites should be convenient and trusted such as churches, barbershops, and community sites. While a number of individual and combination efforts have been developed and implemented, data that disentangle components that are the most effective are sparse. This rapid review provides a basis for developing strategic implementation to increase COVID-19 vaccine uptake in this ongoing pandemic and planning to promote health equity for future bio-events and health crises.

## Introduction

Despite the effectiveness of the COVID-19 vaccines, many people in the USA are not fully vaccinated [1]. By August 2021, unvaccinated people were six times more likely than those vaccinated to be infected with COVID-19 and eleven times more likely to have died [2, 3]. Counties with low vaccination rates have higher rates of COVID-19 [4]. Together, these data add evidence that COVID-19 vaccines have been effective at reducing hospitalization and death.

Communities of color continue to have a high burden of COVID-19 cases, hospitalizations, and death. Yet rates of COVID-19 vaccine uptake among Black and Latinx communities have continued to lag behind that of Whites [5]. This disparity centers around multi-level issues of pervasive structural inequities including easy and convenient access to COVID-19 vaccines. The focus for this review is on recommended and implemented approaches to enhance uptake of COVID-19 vaccines specifically for Black communities.

Given the urgency of addressing the disparities in COVID-19 morbidity, mortality, and vaccine uptake among Black people, we undertook a rapid review [6] limited to the first nine months of vaccine distribution in the USA. This review examines a broad range of evidence from academic literature to media reports and perspectives of persons engaged in intervention implementation. For COVID-19 vaccine uptake, we examined three main components (beyond the logistics of product development, distribution, and workforce distribution) to approaches and interventions that can address vaccine hesitancy and uptake. These were framed as questions: (1) what is the content of the message to promote uptake of the vaccine; (2) who delivers the message and provides the service, and (3) where and when is there access to the vaccine services. Given the timeframe of the review, our report considers the relative promise if not effectiveness for intervention strategies that can encourage vaccine uptake among Blacks.

## Methods

Overall methods for this review have been described elsewhere [6]. Specific for this report, the search engines used were as follows: PubMed, Google, medRxiv, and *The New York Times*. Since the data for this report were generated within the first nine months of COVID-19 vaccine roll-out, the decision was made to widen the traditional academically oriented search engines and programmatic and experience-based reports through media accounts. Key terms selected for the searches were as follows: First term: “COVID-19 vaccine”. Second terms included “African-American,” “Black American” OR “Black African American; “intervention” AND “United States”. Third terms included “Community” OR “access” OR “collaboration” OR “outreach”, “intervention”, “hesitancy”, “trust”, “best practices”, “rollout”, “provider recommendation”, “misinformation”, “access”. The same terms were used consistently across platforms. Links within articles were also evaluated when relevant. Searches were conducted in July and August 2021. In order to meet inclusion criteria, reports had to have been published between December 2020 and August 2021 (to coincide with the national roll-out of the COVID-19 vaccines), include original data on the USA, and be written in English.

## Results

Search results were as follows: On PubMed, two of the 45 results yielded met our inclusion criteria. On Google, 59 of the 140 results yielded met our inclusion criteria (excluding repeat articles). None of the 103 results yielded on medRxiv met our inclusion criteria, nor did any of the 19 yielded on *The New York Times*.

### Addressing Mistrust

According to experts, acknowledging and validating Black Americans’ concerns, which are rooted in lived experience and knowledge of historical events, is a key step to overcome vaccine hesitancy [7–10]. Prior to providing information about the vaccine, public health messages and healthcare providers should acknowledge past and contemporary injustices and racism as justifiable reasons for mistrust. Messengers ought to appreciate the significance of the origin of mistrust and encourage vaccination by using autonomy-supportive, empathetic, fact-based, non-confrontational, non-coercive, and non-judgmental communication. Confronting racism and structural inequality in medicine and beyond is paramount to increasing vaccine acceptance [8–13]. This requires engaging in sustained, authentic efforts to increase the trustworthiness of healthcare organizations, pharmaceutical companies, and the government. Four themes emerged in reports on priorities for addressing structural inequality as it pertained specifically to decreasing COVID-19 vaccine hesitancy among Black people. They are (1) increase trust in healthcare providers, (2) increase vaccination site (and general healthcare) access, (3) increase funding for equity-focused initiatives, and (4) create empowered equity task forces.

Addressing mistrust can help to reduce COVID-19 vaccine hesitancy and increase vaccine uptake. Increasing trust in COVID-19 vaccines will require a combination of short- and long-term strategies that use messages well-catered to needs of Black communities delivered through trusted messengers. Trusted communication is required to ensure a thorough grasp of how the safety and effectiveness of COVID-19 vaccinations are. People may lose faith in vaccines and those who provide them if they do not receive clear, consistent, and easily accessible information that supports their decision-making. Trusted communication strategies have been employed to tackle mistrust and misinformation in Black communities. Strategies highlighted by experts that can help to promote trust within Black communities in COVID-19 vaccines were lead with listening, tailor responses to patient concerns, describe the development and regulatory processes of the COVID-19 vaccines, and acknowledge uncertainties [14]. A number of reports go beyond expert opinion and describe specific trusted persons who can serve as communication channels that have been implemented to decrease vaccine hesitancy among Black Americans. As described more fully below, these have included Black healthcare providers, health system leaders, and community partners such as clergy and barbers.

### Trusted Sources and Communication Channels

#### Black Physicians, Healthcare Providers, and Health Leaders

A personal physician is the person most likely to influence decisions on whether to take the vaccine [15]. However, the reach and influence of physicians is limited to those who are connected to an established practice. Many people either do not have regular visits with their physicians, nurse practitioners, or physician assistants. Approaches beyond direct contact with clinicians are needed to influence population vaccine uptake. These include street outreach, social media, partnerships with social influencers, and collaboration with professional organizations.

To reach beyond one’s practice, Black medical leaders and nurses have used social media and community outreach to pass on COVID-19 vaccine information. For example, in Washington D.C., an infectious disease physician worked to build trust in the Black community through engaging individuals in discussion. One listener noted that: “I was emphatic: no’: after my initial skepticism, I was only persuaded over several months… to have a COVID-19 vaccine. [16]” Black physicians and nurses across the nation have also used social media such as YouTube and Instagram to explain COVID-19 vaccine science to the public, dispel related myths, and share their experiences after receiving the vaccine [16–18].

Black physicians have also partnered with government and social influencers to share information nationally. A community-based initiative called Black Coalition Against COVID organized in Washington D.C. has a hosted series of virtual town hall meetings in collaboration with four Black medical schools along with the National Medical Association, the National Black Nurses Association, the Montague Cobb Health Institute, the National Urban League, and Blackdoctor.org [19].They assembled Black healthcare professionals and prominent Black health leaders to share targeted information on the safety and effectiveness of the vaccine with the Black community.

In New England, the president and CEO of Trinity Health organized series of conversations with the Black community through webinars about the COVID-19 vaccine [20].The objective was to build trust by addressing fears related to the vaccine, explaining the vaccine development process, and ultimately increase buy-in to COVID-19 vaccine. The webinars took place weekly on weekday evenings from January into February 2021. About 200 people attended his last session. Before the conversation, 38% of the audience said they would not take the vaccine, but after the conversation, many of those who had said they wouldn’t had changed their mind, although the exact figure was not reported [21].

In Maryland, the president of the University of Maryland Baltimore County and his wife participated in a phase 3 clinical trial to show that Black people have been involved in the development of the vaccine. In an interview, he reported that by taking part to the clinical trial, he wanted to change attitudes about the vaccine in the Black community, save lives, and encourage people to believe in the science behind them. As of February 2021, Black or African American individuals represented at least 11% of participants in Pfizer-BioNTech vaccine studies. Black participants in these trials have a right to expect and trust that whenever vaccinations are accessible, Black communities will have equitable access to them. In addition, participation to COVID-19 vaccine clinic trials by Black leaders is a way to encourage people to believe to science and get the vaccine before it becomes available [21].

#### Community Partners

Current evidence suggests community involvement is crucial to address the imbalanced prevalence and mortality of COVID-19 in Black communities, and the manifestations of long-standing structural and social inequality. Public health experts have noted that community organizations and networks need to be involved in each step of vaccination education and distribution programming to optimize uptake within the community [22].Faith-based institutions and respected community influencers have been noted in the literature as trusted and important partners in reaching Black communities. The important role of community partners in creating satellite vaccination sites cannot be understated, and will be described in detail in a later section on improving vaccine access.

Black faith-based leaders have engaged in a variety of outreach vaccination campaigns. Pastors in South Carolina led a campaign to increase awareness of the contributions of Black scientists such as Kizzmekia Corbett in the development of vaccine candidates in order to increase trust in the safety of the vaccines [23].In Florida, a pastor of Tallahassee’s Bethel Missionary Baptist Church convened a statewide COVID-19 Vaccine Community Engagement Task Force that included historically Black college and university representatives, business owners, media representatives, and politicians. They worked with the state government to get vaccinations to Tallahassee’s Black communities and underserved neighborhoods [24].The International Vaccine Access Center at Johns Hopkins University has collaborated with faith leaders to create a national marketing campaign, combined with outreach by Black doctors and other respected community influencers to promote vaccination [25].

Barbershops have been noted by experts as potentially key community influencers based on their ability within Black communities to address health crises in the past. Although impact was not reported, there has been an ongoing vaccine promotion effort by Randolph’s, an African barber in a Washington D.C. suburb [26].The intervention consisted of discussions with each client coming to the barbershop about the benefit of taking the vaccine and the risk of not taking it. Shops like Randolph’s help to share accurate information about vaccines with their customers, even hosting on-site vaccination events in partnership with local healthcare providers. “It’s familiar… not an office… I feel comfortable getting the shot here,” reported a client. At the national level, the Biden administration subsequently teamed up in June 2021 with several organizations, including the Black Coalition Against COVID, to launch an initiative called “Shots at the Shop,” to encourage Black-owned barbershops and beauty salons to promote vaccine education and outreach on a local level [27].

Community centers, non-profits, and community care clinics have also worked to decrease hesitancy. For example, the Minnesota Community Care Center meets people where they are to have conversations with them about cover the spectrum of vaccine hesitancy, acceptance, trust, and possible side effects that might occur after the second dose. A partnership between Minnesota’s Community Care and University Health Care Centers has allowed staff to create videos and flyers to dispel COVID-19 myths to give testimonials about the vaccine. Rotating presentation slides in the clinic building going over vaccine facts, and deliberate effort has been made to get information in front of population [23].

During the period after these outreach campaigns have launched, there are ecological data to suggest temporal changes in attitudes about the vaccine among Blacks. A poll from The Associated Press in late March 2021 found that ~ 24% of Black American said they would not get vaccinated, down from 41% in January. In April 2021, the proportion of Blacks willing to get the vaccine rose to become the same as the proportion of Whites at 26% and Hispanics at 22%. In an interview with the Associated Press, the Executive Director of the American Public Health Association recognized that attitudes toward the vaccine among Blacks had taken “almost a 180-degree turnaround” by April 2021 as outreach campaigns have worked to combat misinformation [24].

### Addressing Current Structural Inequalities in Healthcare

#### Improve Equitable Access to Healthcare

Equitable access to health services including vaccination sites is needed to ensure that Black communities are able to receive the available COVID-19 vaccines [8, 10, 12, 28].Due in large part to enduring structural racism spanning from urban planning to access to health insurance, Blacks are less likely than Whites to live near medical health clinics and these medical deserts pose an issue in accessing traditional vaccination sites [29].To address this structural issue, vaccines should be made available in a variety of community settings that are trusted, safe, and accessible to the diverse array of individuals that make up Black communities. More information on pop-up and mobile clinics can be found below. Provisions ought to also be made for meeting the needs of hard-to-reach populations that Blacks are over-represented in, such as prisons and homeless shelters. An example of this can be found in Minnesota where the Community Care clinic, a large federally qualified health center with shelter-based, and street-outreach clinics serving homeless populations were mobilized to provide COVID-19 vaccines [23].

In addition to attending to location-related needs, clinics should also be made available at accessible times for those unable to take time off work. Rather than only having piece-meal programs to increase access for Black communities on the local or state level, some experts have identified the need for a national coordinated strategy [30].Creating systems that allow for increased healthcare access beyond just COVID-19 vaccination is an important step in creating actual and perceived structural equity in American medicine, which can increase the likelihood that Blacks see the system as meaningfully invested in their well-being as a community and therefore more likely to trust it. At the national level, the Biden administration began acting on a January 2021 pledge to build new medical facilities and temporary mobile clinics in underserved Black communities [11, 31].

#### Producing More Black Health Professionals

As doctors have been widely cited across demographic groups as a reputable source for advice regarding vaccinations [32], it is important that efforts are made to ensure that this source can be optimized for use in the Black American population [33].Experts have urged to increase the number of Black physicians [11, 34].Evidence has been reported that demonstrating greater trust by Black persons in clinical interactions is characterized by racial concordance and this can be used to establish greater trust in the medical system as a whole [7, 11].This requires addressing the widely reported hostile training environments, inadequate financial support, and lack of outreach and mentorship options by Black students interested in becoming healthcare professionals [7, 35].

Other strategies include training doctors of all demographics to take a more participatory style in seeing minority patients and empowering Black patients through education and support from community health workers to advocate for themselves during clinical appointments. Trainings of this sort have been implemented by the Director of the Johns Hopkins Center for Health Equity [36].In passing, many persons do not have access to a physician for routine visits and when they do, the physician is under pressure for brief visits to remain financially viable. New York State has instituted payment to clinicians for time spent on working with patients to encourage uptake of the vaccine as an incentive to include this in regular patient visit [37].Also, there has been underutilization of nurse practitioners, physician assistants, and pharmacists who can be influential. Gallup polls over the past 19 years have shown nurses to be the most trusted profession, ranking higher than physicians [38], yet the most recent Woodhull Study showed nurses to be invisible in the media [39].

#### Fund Equity Building Initiatives

It is crucial that legislative bodies expand funding to support and strengthen national, state, and local efforts on equitable and effective COVID-19 vaccination planning, communications, distribution, and administration [22, 28].This must include funding to Black-serving institutions to support vaccine distribution at the local level by Black-serving institutions. To address the impacts of structural racism and correct for its ongoing harm, it is important for funding to be allocated toward groups created specifically for meeting the manifold needs of Black persons beyond just vaccinations. This is especially necessary as the COVID-19 pandemic has disproportionately impacted the Black community in terms of morbidity and mortality, stretching thin the limited capacity of community-led groups. If these groups are expected to pick up where the traditional healthcare system has fallen short in its mandate to serve all Americans, they need to be provided with the means to do so. The Michigan State government has been referenced as a promising example on this point as their vaccine equity task force has solicited applications to fund numerous promising initiatives from community organizations [40]. As another example, the Massachusetts State budget 2021 calls for public–private partnership for building equity [41].

#### Create Effective and Empowered Equity Task Forces

States and localities should create COVID-19 vaccine equity task forces with clear objectives and specific success metrics that maximize communication and collaboration between other related equity task forces [40].There have been equity task forces created across the country. At the national level, a federal health equity task force has been meeting to provide guidance to states [42, 43].

At the state level, New York’s Vaccine Equity Task Force was launched on December 21, 2020. It is chaired by Secretary of State Rossana Rosado, Attorney General Letitia James, National Urban League President & CEO Marc Morial, and Healthfirst President & CEO Pat Wang [44]. The New York State Vaccine Equity Task Force has worked statewide with Black churches, public housing, and community centers to run a public education campaign targeting the Black communities [11].The Maryland Vaccine Equity Task Force [45], led by Maryland National Guard Brigadier General Janeen Birckhead, is working with the state’s 24 local health departments to focus COVID-19 vaccination efforts on underserved, vulnerable, and hard-to-reach populations to ensure the equitable delivery of vaccines.

The Michigan Task Force on Racial Disparities was created in April 2020 per an Executive Order to address COVID-19 disparities [46].The Task Force advises the Governor and investigate the causes of COVID-19-related racial disparities and recommend actions to take to combat them. The Task Force includes the lieutenant governor, the director of the Department of Health and Human Services, the chief medical executive, and 24 members appointed by the governor reflecting the diverse geographic, economic, racial, cultural, gender, and occupational composition of this state. The Task Force helped guide targeted media messages, prioritized and expanded testing resources, supported worker quarantine, distributed free masks and healthy food boxes, and provided equity training to healthcare providers to address health equity. To implement this guidance, the Michigan Health and Human Services (MHHS) partnered with other organizations across the state to understand the needs of all communities including Black communities to address vaccine misinformation by building relationships with community leaders, facilitating town halls to discuss vaccine issues. It also built on the partnerships and knowledge from other equity task forces, and in addition to empowering community organizations, it aided with guiding targeted media messages, prioritizing, and expanding testing resources for Black communities. It also supported worker quarantining, distributed free masks and healthy food boxes, created mandatory bias training for healthcare providers, and used innovative assessment tools to measure their success [23].

### Combating Misinformation

Misinformation plays a significant role in perpetuating vaccine hesitancy within Black communities, and it must be addressed to increase widespread vaccine uptake. The history and valid personal experiences of Black persons contribute to a fear of the government and medical centers, which magnify the power of misinformation [11].It is important that institutions and individuals working to address misinformation in Black communities communicate through trusted sources using tailored, multimodal, and culturally intelligent messaging [7–10, 18, 20].It is appropriate for emphasis to be placed on activities such as fact-finding, rumor control, dispelling of myths and misinformation, message monitoring, and frequent utilization of a variety of spokespersons and audio and visual public service announcements. Developing simple toolkits (e.g., with survey or focus group questions) that can guide communities in assessing specific misinformation-related issues in a feasible and low-cost manner can enable institutions to better address community-specific predictors of hesitancy. It is also crucial to partner with informal and formal Black community leaders to identify trusted information sources, main concerns, and preferred ways to receive the vaccine, for the community by community. Furthermore, leading with empathy is crucial as when people who are experiencing hesitancy because of misinformation are treated with respect rather than contempt, it is more likely that with the right information they can be made to feel a sense of autonomy over their health and health decisions while also following public health guidance.

#### Town Halls and Community Discussions

A prominent strategy noted in the literature to address vaccine hesitancy and combat misinformation within Black communities was hosting community discussions, town halls, and info sessions virtually or in-person. There were a variety of topics discussed, modes of delivery, and hosting entities for these events. Three sources provided some results or feedback. The town hall reported above in addition to two statewide for Ohio and Maryland, one regionally in California and two locally within Maryland and California, all remain accessible nationally online.

The town halls described earlier hosted by the Black Coalition Against COVID used question and answer periods to dispel myths and address specific concerns. The Ohio Department of Health and the Ohio Department of Mental Health and Addiction Service held a series of virtual town halls with medical experts, community leaders, and public health professionals to address common concerns in Black communities in February of 2021 [47]. The sessions were livestreamed on Facebook, YouTube, and the state government website and later televised on local stations throughout the state.

The University of Maryland Medical System and the Health Advocates In-Reach and Research Network of Prince Georges County, Maryland (a collaboration between Black barbershops and public health practitioners), both held sessions in January of 2021 [48].In addition to creating event recordings available online, the University System also publicized a write-up of key questions and answers from the session.

In California, a community-academic partnership in Inland Empire—a racially diverse, Democrat metropolitan region in southern California—hosted free virtual, dialogue-based, live, interactive webinars on the fears and facts associated with the COVID-19 vaccine on a monthly basis beginning in December 2020 [49].The partnership included health faculty at a local university, two large associations of faith-based organizations, and a cultural community health outreach program, and all webinars were advertised on institutional and organizational social media pages. The webinars impressed upon audiences of 500 live participants (and 7000 total viewers) the urgency of the situation by describing health disparities, empathized with concerns associated with the COVID-19 vaccine, and shared personal stories of overcoming hesitancy. A town hall hosted by the University of Southern California in Los Angeles in February 2021 featured top medical experts in the state dispelling misinformation and addressing pressing questions regarding vaccine safety, efficacy, and access [50].This event garnered 200 views, and some viewers reported to the host that they chose to schedule their vaccine appointment after the session.

#### Public Education Campaigns, Grassroots Initiatives, Hotlines, and Informal Surveillance

Implemented strategies to decrease the effect of COVID-19-related misinformation in the African American community have taken a wide variety of forms and scales, none of which provided data on their impact. The New York State Vaccine Equity Task Force has worked statewide with Black churches, public housing, and community centers to run a public education campaign targeting the Black communities [11].In Travis County, TX, the county constable has led a community outreach team to host pop-up events to answer vaccine-related questions in addition to making 2000 phone calls, visiting 2500 apartment complexes and mobile homes, and sending 2000 text messages [51]. There have also been efforts to address misinformation-related concerns across the nation using online initiatives. The Kaiser Family Foundation partnered with historically Black colleges to create a series of videos addressing common misconceptions and misinformation tropes regarding the COVID-19 vaccine and a family physician in Orlando uses her influence on social media to expel COVID-19 vaccine–related myths [17, 18].

There have also been local efforts to combat misinformation recorded in communities within Minnesota and Texas. Twin Cities is a metropolitan area home to one of the largest populations of Somali American. A multipronged grassroots community response group (made up of health workers) created an informal hotline in June 2020 for Somali Americans in the community to help with COVID-19-related questions or issues and when vaccines became available, with vaccine-related concerns, with questions regarding COVID-19 vaccinations, or issues navigating their experiences with COVID-19 [23].This hotline was publicized using word-of-mouth and Somali-American media spots. Community-based organizations have also well-suited to serve as signal detection systems for monitoring trends in misinformation and disinformation [10]. Misinformation abounds [, 52, 53]. The *National Black Church Initiative*, [54] a network of 150,000 churches for example, has used its influence to act as an informal quasi-emergency alert system identifying and addressing prominent misinformation trends related to COVID-19 within their respective communities [18].

### Increasing Access to COVID-19 Vaccines: Addressing Barriers to Convenience

#### Non-traditional “pop-up” Settings and Assistance with Transportation

As noted earlier, personal physicians are seen as the most trusted source of information about COVID-19 vaccines. Overall, there are many persons who do not engage in regular medical care, or the frequency of their visits is too far apart to be accessible for information and vaccine in a timely manner. The history and valid personal experiences of Black persons that contribute to a fear of the government and medical centers can contribute to a wariness toward health systems and delay if not avoid healthcare encounters [11].Another challenge is that many physicians have had to prioritize taking care of patients with COVID-19 so that time is limited. Time spent counseling lengthens patient care visits which have not been remunerated. Moreover, COVID-19 vaccine deserts have been mapped where Blacks had further distances to drive than Whites to receive services [55].Transportation, including the logistical gymnastics required for people without a car or who rely on public transportation, can led to differential access to COVID-19 vaccine [56].

Some of the non-traditional strategies to ensure equitable access to COVID 19 vaccine could include providing vaccine in mass vaccination clinics or outdoor parking lots, community centers, and spaces (e.g., barbershops/salons, grocery stores, corner stores, recreational centers or courts, YMCAs, Boys and Girls Club); faith-based institutions (e.g., churches, mosques, synagogues); schools and other educational institutions (e.g., local schools), locations where community members can already access other social or community services, pharmacies in the Federal Retail Pharmacy Program, including local/independent pharmacies, local health clinics/centers or Federally Qualified Health Centers, mobile clinics, or temporary/off-site clinics (e.g., mobile vans, ambulance services); and employers where community members work [57].

In New York, a regional health system made a concerted effort to reach out to the communities developing “pop-up” sites. Stony Brook University’s Health System’s outreach programs have delivered 350,000 doses of COVID-19 vaccine in Long Island. To serve its patients across the island, SBM worked with the state health department to successfully develop community PODs (i.e., public and private locations that have agreed to dispense medications, generally during a public health emergency, as pop-up sites in underserved communities on Long Island, to reach communities of color and the elderly, as well as help build trust [58].

Michigan State health officials decided to eliminate differences in drive times to ensure there are no racial and ethnicity disparities in vaccination rates. They set that no Michigander should drive more than 20 minutes to reach a vaccination site. Officials set mobile vans on a corner 20 minutes to the senior center in both cities and outlying areas [56].

In New Jersey and California, local governments have attempted to address barriers to accessibility [59,60]. New Jersey has striven to build more easily accessible vaccination sites, focusing on communities with high minority populations and hosting pop-up clinics in collaboration with houses of worship and other local organizations. In California, transportation options include mobile clinics/pop-up sites that are within walking distance, facilitated public transportation, “Call the Car”—plans which allow transportation to and from vaccine appointment to local provider or pharmacy. [61] Formal evaluation of the impacts for these initiatives has not been reported.

Washington D.C. launched a door-to-door campaign as a new strategy to vaccinate residents in neighborhoods severely affected by COVID-19. They provided information on how to sign up for vaccinations. The offices would also call seniors that they already knew to provide help in securing appointments, drawing on the lists of those who had enrolled for snow removal assistance. Fairfax County offered free transportation to vaccination sites for some residents who live farther away. Montgomery County has been prioritizing residents from zip codes with high infection rates. Maryland has opened mass vaccination sites on Fridays at Six Flags America in Prince George’s County and at the Baltimore Convention Center. However, the impact of these strategies has not been reported [56]. In Massachusetts, activists worked to create incentives such as “companion appointments” permitting vaccinations for younger residents who accompanied elder seniors to appointments .

Detroit, MI, a city where over three-quarters of all residents are Black and vaccination rates were 5 percentage points lower than the rest of the state (8% vs. 3%) as of January 2021, also made strides to solve transportation access issues. The mayor of Detroit commissioned private companies to provide free transportation to vaccination sites, opened more vaccination appointments, and hired additional operators [11].

#### Reports of Outcomes for Efforts to Address Access

While there are likely many more programs that exist beyond our search strategy, we note that almost all noted here are descriptive. We identified four that provided a measure of outcomes although the data are limited by design and measurement.

Regionally, notable efforts have been made in metropolitan counties. In Travis County (home to Austin), TX, these have included creating vaccination sites in Black churches and community centers which led to more persons of color becoming vaccinated in the spring of 2021 relative to other counties.

In the city of Madison, WI, an initiative led by the University of Wisconsin–Madison School of Pharmacy, Fitchburg Family Pharmacy, and the Boys and Girls Club of Dane County held a series of pop-up vaccine clinics during April and May 2021 that delivered 600 vaccinations [9]. Learning from the lessons coming out of a similar vaccination initiative hosted by the African American Health Network of Dane County (AAHND) in partnership with Dane County Public Health, the Goodman Community Center, the Lussier Community Center, and the Urban League hosted in April 2021, AAHND delivered over two dozen combined education and vaccination programs across Madison from May through August because they found this enabled them to better reach the Black community. One of their community sites delivered 26 vaccinations in one week in May.

In Philadelphia, the Black Doctors COVID-19 Consortium started providing free testing services in churches and community centers in some of the poorest neighborhoods in April 2020. Then, building on the bonds of trust created by the barrier-free testing, the group began distributing the vaccine in February 2021 and enjoyed high levels of patronage [11]. Their series of free testing clinics, including a 24 hr walk-up vaccination site, has since vaccinated 4000 people [13].

## Discussion

Views have been articulated that the COVID-19 vaccine hesitancy among Blacks is related to mistrust of the government, and distrust of the healthcare system [6]. Framing the issue this way conveys a sense that Blacks are the problem, that Blacks just don’t trust the “system”. However, Blacks have very good reasons not to trust the “system.” That is, not trusting the “system” is a healthy response to structural racism. It’s the structural inequalities and structural racism that’s the problem, not the people.

Confronting systemic racism and structural inequality in medicine, government, and beyond is paramount to increasing vaccine acceptance [8, 11–13]. The lack of literature on interventions to increase uptake of COVID-19 vaccine for this rapid review can be seen as another marker of systemic racism. Yet this review offers possible lessons on how to improve vaccine uptake for Black communities moving forward whether in this pandemic or later for other public health campaigns.

Leadership for public health campaigns depends on healthcare professionals. Strategies that promote the setting of primary care offices are built upon the premise that many Americans rely on their primary care providers to care for them, including COVID-19 vaccine education with their recommendation to accept a vaccine. Rigorous vaccine research has examined interventions for healthcare providers to apply within the context of their office or clinic practice setting such as presumptive announcements or motivational interviewing.

Clinic and office-based intervention research is narrow because the proportion of the population with a relationship or even access to such a healthcare provider is insufficient to be compelling as a primary strategy. This is only more so for Black persons. Moreover, from a logistics perspective, primary care clinics are not necessarily a feasible option early in COVID-19 vaccine roll-out, due to the challenges of such factors as maintaining the cold storage temperatures and the time it takes for patient education especially without patient care reimbursement schemes.

This review highlights the wider role of healthcare providers to link more broadly with communities through communication channels such as social media, outreach as providers outside of the traditional settings such as clinics and office looking to add non-traditional sites such as churches, and also becoming equal partners with trusted community leaders. Investment in public health infrastructure that adopts and expands the lessons described here is warranted. The vastness of the needed response is daunting. However, assuming the policy elements get aligned, achieving the scalability needed is achievable. For example, promising efforts have been developed for “prebunking” [62] as a way to anticipate, neutralize, and counter conspiracy messaging. In addition, California has developed a composite health equity metric used to measure case rate and test positivity to inform vaccination allocation [63].

The approaches covered in this rapid review were limited to the first nine months of the COVID-19 vaccine roll-out. The approaches described here are only those that were identified from the rapid review using selected key terms and so this review is not comprehensive. Most of the articles reviewed were from the popular press. Few reports provided rigorous evaluation of effectiveness which is not surprising because the roll-out was urgent and such studies require more time to complete and publish. Thus, offering inferences that might rank elements for effectiveness is premature.

This review offers a perspective of the real-time mobilization which highlights the early views and experiences toward mobilization. What can be considered is that the results here can be used to generate hypotheses to inform short- and long-term actions to achieve health equity during and beyond this pandemic. Health equity must be built more completely into pandemic preparedness plan that includes engaging community leadership with healthcare providers and public health agencies to shape communication and access. This needs more than ad hoc crisis response and requires investment in building and sustaining comprehensive community-wide infrastructure for rapid response. This requires a commitment to sustained, authentic efforts now and beyond this pandemic to increase the trustworthiness of healthcare organizations, pharmaceutical companies, and the government.
